# The modified COVID‐19 Yorkshire Rehabilitation Scale (C19‐YRSm) patient‐reported outcome measure for Long Covid or Post‐COVID‐19 syndrome

**DOI:** 10.1002/jmv.27878

**Published:** 2022-06-01

**Authors:** Manoj Sivan, Nick Preston, Amy Parkin, Sophie Makower, Jeremy Gee, Denise Ross, Rachel Tarrant, Jennifer Davison, Stephen Halpin, Rory J. O'Connor, Mike Horton

**Affiliations:** ^1^ Academic Department of Rehabilitation Medicine University of Leeds Leeds UK; ^2^ National Demonstration Centre of Rehabilitation Medicine Leeds Teaching Hospitals NHS Trust Leeds UK; ^3^ Covid Rehabilitation Service, Leeds Community Healthcare NHS Trust Leeds UK; ^4^ Airedale NHS Foundation Trust Keighley UK

**Keywords:** COVID‐19, instrument, PACS, phenotypes, PROM, SARS‐CoV2, scale, traits

## Abstract

**Background:**

The C19‐YRS is the literature's first condition‐specific, validated scale for patient assessment and monitoring in Post‐COVID‐19 syndrome (PCS). The 22‐item scale's subscales (scores) are symptom severity (0–100), functional disability (0–50), additional symptoms (0–60), and overall health (0–10).

**Objectives:**

This study aimed to test the scale's psychometric properties using Rasch analysis and modify the scale based on analysis findings, emerging information on essential PCS symptoms, and feedback from a working group of patients and professionals.

**Methods:**

Data from 370 PCS patients were assessed using a Rasch Measurement Theory framework to test model fit, local dependency, response category functioning, differential item functioning, targeting, reliability, and unidimensionality. The working group undertook iterative changes to the scale based on the psychometric results and including essential symptoms.

**Results:**

Symptom severity and functional disability subscales showed good targeting and reliability. Post hoc rescoring suggested that a 4‐point response category structure would be more appropriate than an 11‐point response for both subscales. Symptoms with binary responses were placed in the other symptoms subscale. The overall health single‐item subscale remained unchanged.

**Conclusion:**

A 17‐item C19‐YRSm was developed with subscales (scores): symptom severity (0–30), functional disability (0–15), other symptoms (0–25), and overall health (0–10).

## 1 BACKGROUND

Long Covid (LC) is a term coined by patients. It refers to persistent symptoms 4 weeks after contracting COVID‐19.^1^ Ongoing symptomatic COVID‐19 and post‐COVID‐19 syndrome (PCS) are the scientific terms for symptoms 4–12 and >12 weeks after the illness, respectively.^2^ PCS affects more than two million individuals in the UK alone and more than 50 million cases worldwide.^3^ More than 200 symptoms across 10 organ systems have been reported. The most common symptoms are breathlessness, fatigue, palpitations, dizziness, pain, brain fog (cognitive problems), anxiety, depression, posttraumatic stress, skin rash, and allergic reactions.^4^ It can be a remitting and relapsing condition with a prolonged course causing significant distress and disability in some individuals.^5^

A multidisciplinary team (MDT) of rehabilitation professionals working with patients recovering from COVID‐19 during the first wave of the pandemic developed the original version of C19‐YRS.^6^, ^7^, ^8^ The content was based on staff experience in managing these patients, knowledge from our systematic review of previous outbreaks, and feedback on the scale from patients and healthcare professionals.^7^, ^8^, ^9^ The content was decided using a consensus method. The scale was kept balanced in terms of questions spanning all aspects of 2001 WHO International Classification of Functioning, Disability and Health (ICF) framework.^10^ The scale's content validity was supported by studies^11^, ^12^ using the scale, which revealed symptoms and functional problems similar to other PCS studies reported in the literature.^13^, ^14^

C‐19 YRS was the first validated scale reported in the literature to capture PCS symptoms and grade the severity of symptoms and functional disability in PCS. The scale has been recommended in the NHS England Clinical Guidance for PCS services and NICE rapid guidelines.^2^, ^15^ The scale has been translated to numerous international languages and is currently used in many PCS studies worldwide. There is also a digital format of the scale available where the patient can complete the questionnaire on a smartphone application; the clinician can access the results on a web portal; both the patient and the clinician can use the system to monitor progress and response to ongoing treatments for PCS.^8^

The original C19‐YRS is a 22‐item patient‐reported outcome measure (PROM). Each item is rated on a 0–10 numerical rating scale, where 0 represents symptom not present, and 10 illustrates symptom being extremely severe or life disturbing. The C19‐YRS has four subscales concerned with the severity of patients' key symptoms, functional limitations, overall health, and additional symptoms. The scale also captures pre‐COVID scores for comparison.^8^ Questions 1–10 form the symptom severity subscale (score 0–100), Questions 11–15 the functional disability subscale (0–50), Question 16 is the overall health score (0–10), and Questions 17–22 the additional symptoms subscale (0–60).^16^ The classical psychometric analysis of the C19‐YRS in a sample of 188 PCS patients showed good data quality, good scaling and targeting, and high internal consistency (Cronbach's *α* = 0.891), with good reliability of individual subscales.^16^ Some items were identified as having poor scaling assumptions and targeting, such as swallowing, incontinence, fever, and skin rash. It was determined that the contribution of these items to the overall measurement properties of the scale was limited.^16^

Although the classical psychometric analysis of the C19‐YRS was promising, a further analysis using modern psychometric approaches (Rasch analysis) was included as part of the C19‐YRS development plan. The Rasch model^17^ is a unidimensional measurement model that satisfies the assumptions of fundamental measurement,^18^, ^19^ meaning it provides a measurement template against which scales can be tested. Rasch Measurement Theory (RMT) provides a way to assess the validity of multi‐item latent scales where the items (questions) are summed together to form an overall total score. RMT provides a unified framework for several aspects of internal construct validity to be assessed, where it can highlight measurement anomalies within an item set. It should be emphasized that this C19‐YRS development phase was intended to identify any specific measurement issues that would inform the development of a psychometrically robust modified version of the C19‐YRS.

Since the development of the original C19‐YRS scale early on in the first wave of the SARS‐CoV‐2 pandemic, essential symptoms, such as postexertional malaise (PEM), have been identified as clinically crucial in the management of Long Covid. Such symptoms, mainly those identified as necessary by patients and healthcare professionals, needed to be considered for inclusion in the modified version of the scale.

Therefore, this study aimed to test the psychometric properties of the scale based on the Rasch model, and create a modified version of the C19‐YRS that optimizes the measurement characteristics of the scale while incorporating essential insights from both patients and healthcare professionals.

## 2 METHODS

### 2.1 Rasch analysis

Rasch analysis was completed with RUMM2030 software,^20^ and carried out separately for the C19‐YRS symptom severity subscale (10 items) and the functional disability subscale (5 items). The overall health score comprises a single item, which is treated independently from the other subscales and is therefore inappropriate for Rasch analysis. The additional symptoms subscale was not assessed, as these items provide supplementary information to the clinical staff rather than contributing to the symptom severity subscale.

Several tests of fit were carried out at the scale level and the item level; these are all described in more detail elsewhere.^21^ All items were assessed for individual fit to the Rasch model relative to the subscale item set; this tests whether each item contributes to the same underlying construct. Misfit was indicated where items were significant at a Bonferroni‐adjusted *χ* ^2^ *p *value or standardized (*z*‐score) fit‐residuals fall outside ±2.5. Tests of local dependency (LD) were carried out to determine whether the response to any item directly impacts any other item in the subscale; LD was indicated using a residual correlation (Q3 value) criterion cut point of 0.2 above average residual correlation.^22^ Response category functioning was assessed to determine whether the response structure of the items worked as intended. A functional 0–10 response category structure for each item would be indicated by sequential response thresholds (the crossover points between adjacent response categories) on the underlying logit scale.^23^ Item bias was assessed through uniform and nonuniform differential item functioning (DIF) testing by sex, age group, disease duration, and hospitalization status; with significant DIF indicated at a Bonferroni‐adjusted analysis of variance (ANOVA) *p *value. Scale targeting was assessed graphically through the relative distribution of item and person locations. Unidimensionality was evaluated by a series of *t*‐tests,^24^ with multidimensionality indicated when independent subsets of items delivered significantly different person estimates, and the lower bound 95% CI percentage of significantly different *t*‐tests was >5%.

### 2.2 Working group

A working group comprising five individuals with PCS, one dietitian, one psychologist, four physiotherapists, two occupational therapists, two rehabilitation physicians, two researchers, and a psychometrician discussed the proposed amendments to the scale. The emphasis remained on keeping the scale as brief and comprehensive as possible without undue burden on the respondent.

## 3 RESULTS

### 3.1 Sample

Data from 370 patients who completed the C19‐YRS scale in a community Long Covid service were collected. The key demographics of the sample are presented in Table 1.

**Table 1:** Demographics of participants

	All^a^	Non‐hospitalized	Hospitalized
	(*n *= 370)	(*n *= 301)	(*n *= 67)
Female (%)	237 (64%)	206 (68%)	30 (45%)
Mean age (years) (SD)	47 (14)	46 (13)	53 (14)
Mean weight (kg) (SD)	82 (22)	80 (21)	92 (22)
Mean BMI (kg/m^2^) (SD)	29 (7)	28 (7)	32 (7)
Ethnicity (%)			
White	303 (82%)	250 (83%)	52 (78%)
Black	7 (2%)	4 (1%)	3 (5%)
Asian	36 (10%)	25 (8%)	10 (15%)
Mixed/Other	18 (5%)	17 (6%)	1 (2%)
Smoking status (%)			
Never smoked	235 (64%)	198 (66%)	37 (55%)
Current smoker	24 (7%)	21 (7%)	2 (3%)
Ex‐smoker	105 (29%)	77 (26%)	27 (40%)
Admitted to hospital (%)	67 (18%)	0 (0%)	67 (100%)
Median duration of symptoms (weeks) (IQR)	30 (21‐51)	33 (22‐51)	25 (18‐45)

^a^ Where numbers do not total 370, this is due to missing data

### 3.2 Rasch analysis

#### 3.2.1 Symptom scale

Initially, 12 items were entered into the Rasch analysis. The “breathlessness” section comprises three separate items (breathlessness at rest; breathlessness on dressing yourself; and breathlessness on walking up a flight of flight stairs). These three breathlessness items displayed a significant degree of dependency (pairwise Q3 value 0.57 between breathlessness 1 and breathlessness 2; pairwise Q3 value 0.53 between breathlessness 2 and breathlessness 3; Q3 criterion value indicating dependency = 0.12). Although this finding makes complete conceptual sense, it also means that the three separate items should not all be included in contribution to the total score of the symptom severity scale. Therefore, the breathlessness section was reconfigured so that only the maximum score observed across the three items was used, resulting in a single maximum breathlessness item.

**Figure 1:**
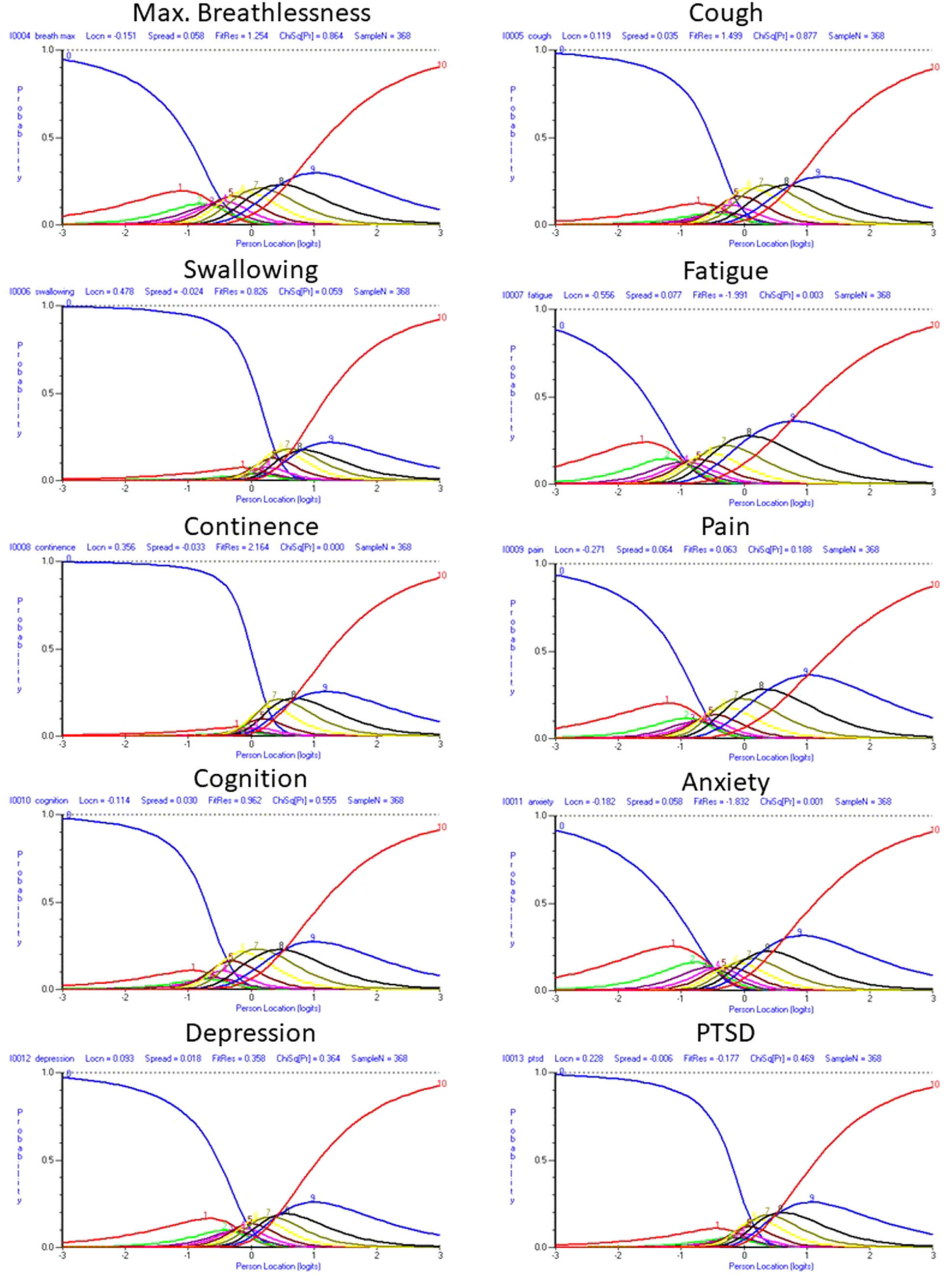
Response category probability curves for each item of the original C19‐YRS symptom severity subscale, with 0–10 response structure.

Initial Rasch analysis of the Symptom Severity scale (10 items, including a single maximum breathlessness item) looked promising but revealed certain measurement issues with the item set. Overall scale fit statistics are presented in Table 2. Three items displayed misfit on the *χ* ^2^ statistic (fatigue, continence, anxiety), with the continence item displaying the largest degree of misfit. Four pairwise dependencies were identified. Listed in order of magnitude (Q3 criterion = 0.10), these were between: anxiety & depression (Q3 = 0.38); fatigue & cognition (Q3 = 0.22); anxiety & post=traumatic stress (Q3 = 0.16); cough & swallowing (Q3 = 0.14). On testing the functioning of the response categories, all items displayed reverse thresholds. It was apparent that a 0–10 response structure was inappropriate for these items, as a logical progression of ordered response thresholds was not observed for any of the items (see Figure 1). The extent of the disordering was variable depending on the nature and content of the item, with the continence and post‐traumatic stress items particularly unsuited to this response structure.

**Table 2:** Rasch analysis summary statistics of C19‐YRS subscales

			Item fit residual		Person fit residual		Overall *χ* ^2^ interaction					Unidimensionality *T*‐tests (CI)	
Analysis	Number of items	Valid *n* (no. of extremes)	Mean	SD	Mean	SD	Value	*df*	*p*	PSI	Alpha	Proportion significant	CI
*Symptom severity subscale*													
Initial	10	368 (1)	0.31	1.36	−0.20	0.99	97.6	50	<0.001	0.80	0.82	0.106	0.084–0.129
Rescored	10	368 (1)	−0.05	1.33	−0.24	1.03	78.5	50	0.006	0.78	0.80	0.071	0.049–0.093
Minus anxiety	9	368 (1)	0.02	0.90	−0.24	0.96	47.8	45	0.360	0.74	0.76	0.06	0.038–0.082
Minus depression	9	368 (1)	−0.05	1.06	−0.26	1.00	61.2	45	0.050	0.76	0.77	0.06	0.038–0.082
*Functional disability subscale*													
Initial	5	350 (18)	−0.08	1.77	−0.34	0.99	48.9	25	0.003	0.75	0.80	0.026^a^	0.003–0.049
Rescored	5	349 (19)	0.20	1.37	−0.31	0.99	42.9	25	0.014	0.72	0.77	0.026^b^	0.003–0.049
Target values			0	1	0	1	Nonsignificant			>0.7	>0.7	Lower CI < 0.05	

Abbreviations: CI, confidence intervals; df, degrees of freedom; extremes, people scoring either maximally or minimally across the complete item set; PSI,  Person separation index.

^a^ Limited power in unidimensionality *t*‐test.

^b^ Low power in unidimensionality *t*‐test.

#### 3.2.2 Rescoring

**Figure 2:**
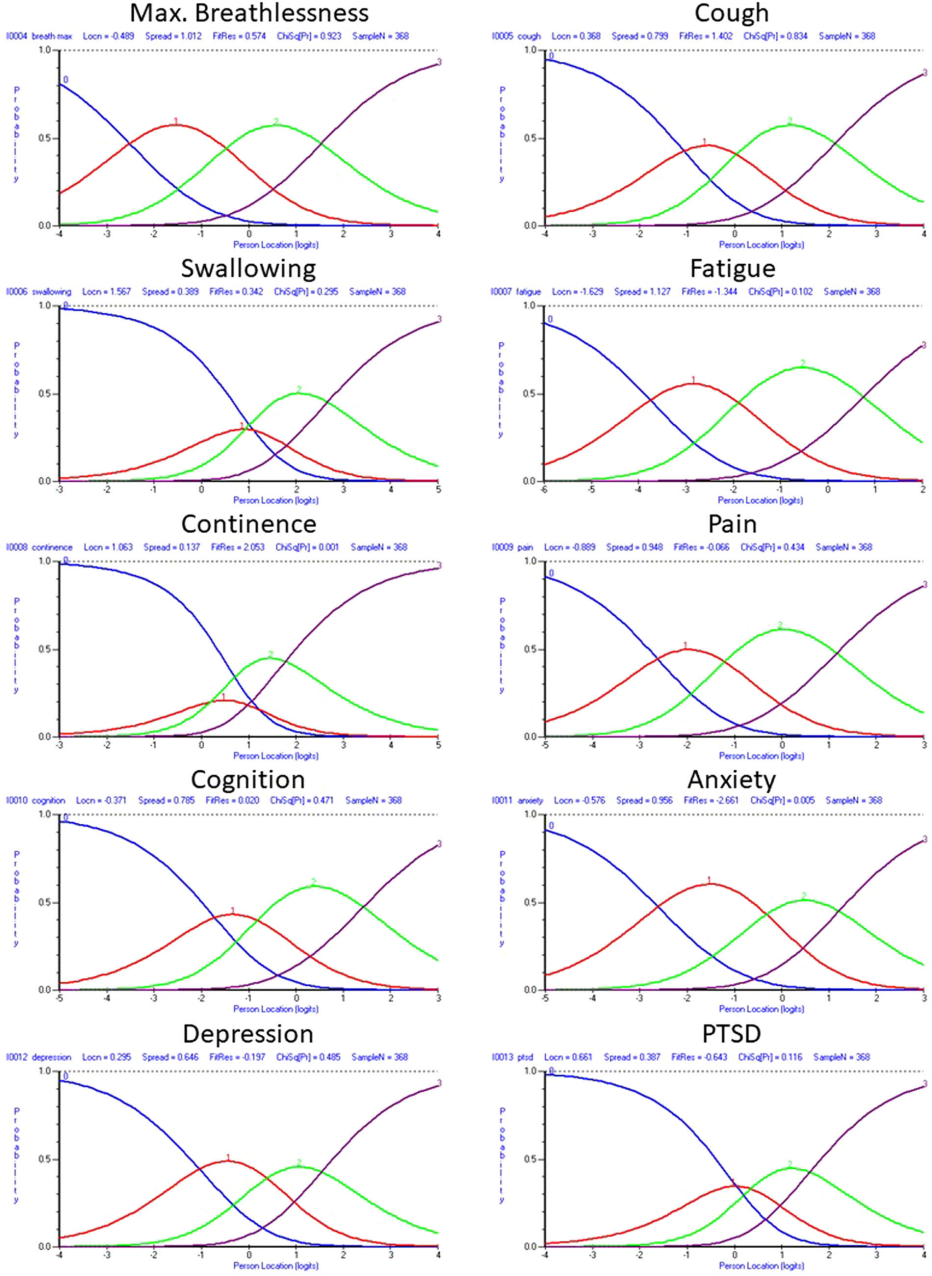
Response category probability curves for each item of the symptom severity subscale, with rescored (implied) four‐point (0–3) response structure of the modified C19‐YRS.

The inappropriate response structure was discussed within the working group. It was decided that four response options would seem a reasonable alternative, striking a balance between the number of measurement points and the amount of conceptually different, distinguishable response categories. Various post hoc rescore options were tested, with the most appropriate 4‐response alternative applied. This rescore was: 0 (no problem); 1–5 (mild problem/does not affect daily life); 6–8 (moderate problem/affects daily life to a certain extent); 9–10 (severe problem/affects all aspects of daily life/life‐disturbing). It should be noted that this scoring structure was applied post hoc to the 0–10 scoring system and that this rescoring is only implied; respondents have not yet been presented with this 4‐category response structure. This new response structure improved the threshold ordering across all items, although the swallowing and continence items still displayed slight disordering (see Figure 2). These items appear more suited for dichotomous or binary (yes/no) response categories.

**Figure 3:**
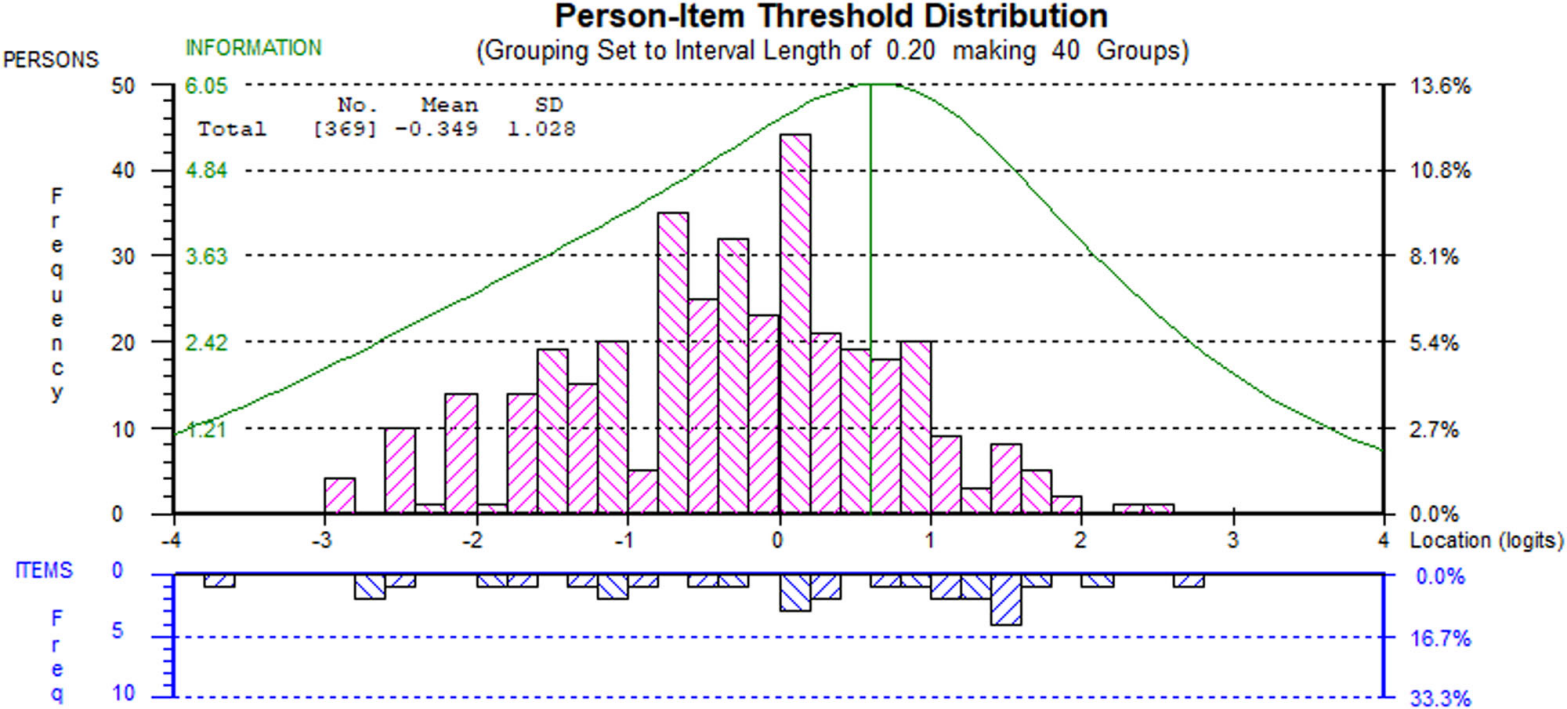
Scale‐sample targeting of symptom severity scale.

Overall scale fit statistics following rescoring are presented in Table 2. At this point, two items still displayed misfit on the *χ* ^2^ statistic (continence, anxiety), with the anxiety item also showing a fit residual of −2.66. The rescoring had little effect on the pairwise dependencies, which remained present as previously reported, and the scale‐sample targeting was good (see Figure 3). There was no DIF by sex, age group, or disease duration group. However, a uniform DIF by hospitalization status was observed for the PTSD item, with hospitalized patients having higher expected PTSD values than nonhospitalized patients.

Also, although it was not the intention of the study to determine this, distributional differences between certain demographic groups were observed. Significant score differences by sex (females more severely affected than males, *p* = 0.02), age group (people aged 50+ more severely affected than those below 50, *p* < 0.01), hospitalization status (hospitalized people more severely affected than those not hospitalized, *p* < 0.005), and BMI group (underweight group more severely affected than overweight, who are more severely affected than healthy weight, *p* < 0.001) were observed.

Further exploratory procedures suggested that the apparent dependency impacted the overall fit of the scale, as removal of either the depression item or the anxiety item resulted in a well‐fitting, unidimensional scale (see Table 2). The dependency suggests that using a single item score for these two symptoms will work better than using separate scores from dependent items.

#### 3.2.3 Functional disability scale

Initial Rasch analysis of the functional disability scale (5 items) looked promising but revealed specific measurement issues with the item set. Overall scale fit statistics are presented in Table 2. At this point, only one item was borderline misfitting on the *χ* ^2^ statistic (ADL). A pairwise dependency was observed between mobility & personal care (Q3 = 0.06; Q3 criterion = −0.03). Again, as with the symptom severity scale, the most substantial issue appeared to be the functioning of the response categories, where all items except activities of daily living (ADL) displayed reverse thresholds (not presented). It became apparent that a 0–10 response structure is inappropriate for this item set. Items were rescored in the same 0–4 manner as the symptom items. The overall scale fit statistics following rescoring are presented in Table 2.

**Figure 4:**
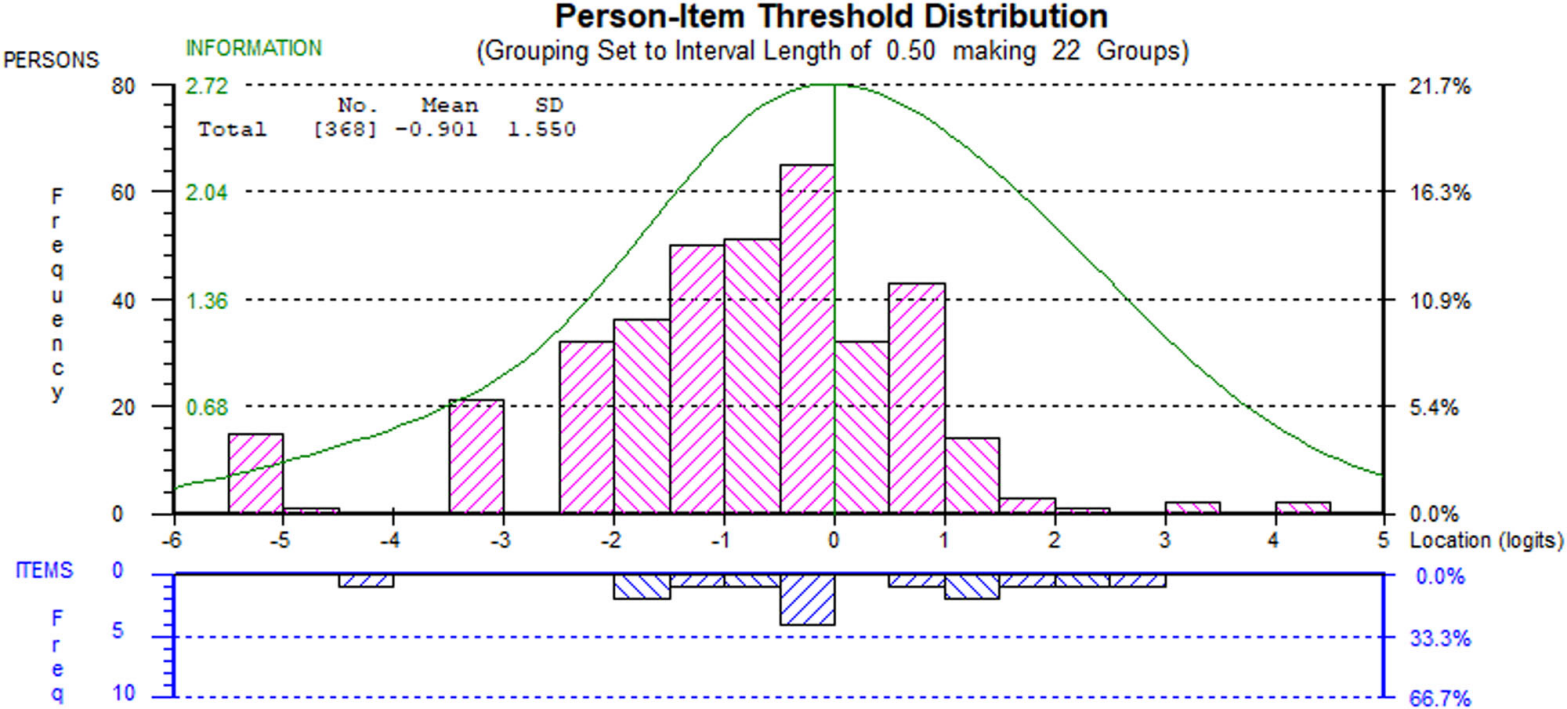
Scale‐sample targeting of the functional disability scale.

At this point, one item still displayed misfit on the *χ* ^2^ statistic (personal care), and the previously observed pairwise dependency between mobility and personal care was still present. There was no DIF by sex, disease duration group, or hospitalization status, although the mobility item does display slight DIF by age. The scale‐sample targeting was good (Figure 4).

As with the symptom severity scale, distributional differences between demographic groups were observed, with mean score differences by sex (females more severely affected than males, *p* = 0.02), age group (people aged 50+ more severely affected than those below 50, *p* < 0.01), hospitalization status (hospitalized people more severely affected than those not hospitalized, *p* < 0.005), and BMI group (underweight group more severely affected than overweight, who are more severely affected than healthy weight, *p* < 0.05).

#### 3.2.4 Working group

The working group acknowledged the potential psychometric issues and strengths of the original C19‐YRS. The suggested rescoring structure for the symptom severity and functional disability subscales was supported. Additional items were added to the scale as necessary during the clinical presentation and evolving literature (such as post‐exertional malaise and altered taste and smell sensation). The apparent dependency of the anxiety and depression items was taken into account by including anxiety/mood as a singular contributing item. As the continence item remained problematic in terms of its fit and response structure, this was moved into the additional symptoms subscale, where a binary response structure was utilized. The final list of main symptoms included in the modified C19‐YRS (C19‐YRSm) symptom severity subscale has breathlessness, cough/voice, smell/taste, fatigue, pain/discomfort, cognition, palpitations/dizziness, anxiety/mood/post‐traumatic stress, sleep, and post‐exertional malaise.

The items included in the C19‐YRSm functional disability subscale remained the same, including communication, mobility, personal care, activities of daily living, and social role. The other symptoms subscale includes fever, skin rash, allergies, hair loss, eye changes, bruising/bleeding, visual changes, swallowing, balance, weakness, tinnitus, nausea, dry mouth/ulcers, acid reflux, appetite changes, weight changes, bladder/bowel symptoms, menstrual cycle changes, sleep apnea, and thoughts of self‐harm.

The overall health single‐item subscale was retained in its original 0–10 response structure. Additional information regarding family/carers views and vocational aspects were also included in the C19‐YRSm as they are in the original scale version of the scale. Table 3 lists the critical changes in the modified version of the scale and the reasons for the changes.

**Table 3:** Summary of changes made to the C19‐YRSm (compared to the original C19‐YRS)

	Changes made in C19‐YRSm	Reason for change
Q1–15	Response categories changed from 11 to 4 for each of the items of the symptom severity subscale and functional disability subscale	Rasch analysis suggested disordered thresholds for these items (Figure 1) that improved thresholds post hoc with rescoring (Figure 2)
Q1–10	Provided the four response categories to each of the symptoms within each single item	Working group suggested it would be easier for respondents to rate each symptom rather than rating only the worst symptom (in the original scale). This change would also help those struggling with brain fog to understand and respond to the question
Q4	Capturing altered smell and taste	Working group highlighted the importance of this symptom and emerging evidence on rehabilitation strategies that can be used for these symptoms
Q7	Palpitation and dizziness introduced as a core symptom	Working group suggested that dysautonomia has emerged as one of core mechanisms linked to many of the Long Covid symptoms
Q8	Included post‐exertional malaise as a core symptom	Working group and emerging literature recognized this as one of the characteristic features of Long Covid which explains the fluctuating nature of the condition
Q9	Merged anxiety, mood and post‐traumatic stress in one single item	Rasch analysis showed the local dependence of these items when scored separately (as in the original scale)
Q10	Sleep introduced as a core symptom	Working group suggested to introduce this as one of the key symptoms that characterizes Long Covid and was closely related to fatigue and other symptoms
Other symptoms	Moving swallowing, continence and suicidal idea items to this section	Rasch analysis and working group suggested these symptoms worked more in a dichotomous fashion rather than graded severity of symptom severity scale. Such symptoms with dichotomous responses were placed in the other symptoms section
Other symptoms	Introduction of new symptoms: allergy, hair loss, skin sensation, dry/red eyes, swelling of limbs, bruising/bleeding, visual changes, tinnitus, nausea, acid reflex, appetite, weight changes, sleep apnea, and changes in menstrual cycles or flow	Working group and emerging evidence suggested even though these are not present in all patients they need capturing as these symptoms can be the cause of concern to patients and need addressing by clinicians

## 4 DISCUSSION

The C19‐YRSm captures the severity of the leading persistent symptoms and functional disability in individuals with Long Covid or Post‐COVID‐19 syndrome. Rasch's analysis of the original scale has led to an amendment in the response structure from a 0–10 numeric rating scale to a 0–4 response scale. Other symptoms subscale includes those with a binary response (yes/no). Symptoms that have been added to the original scale reflect inclusion from the evolving literature and feedback from patients and healthcare professionals based on their understanding of the condition and its impact on health.

The new response category structure will be psychometrically beneficial and is more intuitive for patients, with more distinct response categories. Despite the reduction in response categories, there is only a slight reduction in the internal consistency or reliability of the subscales. The improved response structure may enhance monitoring of the condition at different time points and better capture the impact of interventions used to manage the condition.

The scale takes a maximum of 12 min to complete (as reported by the working group) and can be self‐administered by the respondent in various settings (including their home environment). Responding to the items allows the individual to become aware of the various possible presenting symptoms of the condition helping them understand PCS better. After completing the questionnaire, the individual is potentially better positioned to communicate to their family, carer, and clinician about the condition and its impact on their daily lives. C19‐YRSm items and 0–3 response categories have already been adapted for use in the World Health Organisation (WHO) self‐management symptom tracking diary patients use.^25^

The digital format of the C19‐YRSm (available on the ELAROS smartphone application) allows users to track their condition in time and provides them with a visual quantitative assessment of improvement or deterioration of PCS; this is crucial in the management given less frequent face to face contact during the pandemic. Clinicians can monitor the patient's progress using the web‐based clinical portal, and healthcare services can evaluate treatment programmes using the ELAROS digital system. National and international comparison of PCS data (using the paper or digital format of the scale) can be undertaken while assessing the influence of individual demographics and illness characteristics on PCS symptoms.

**Figure 5:**
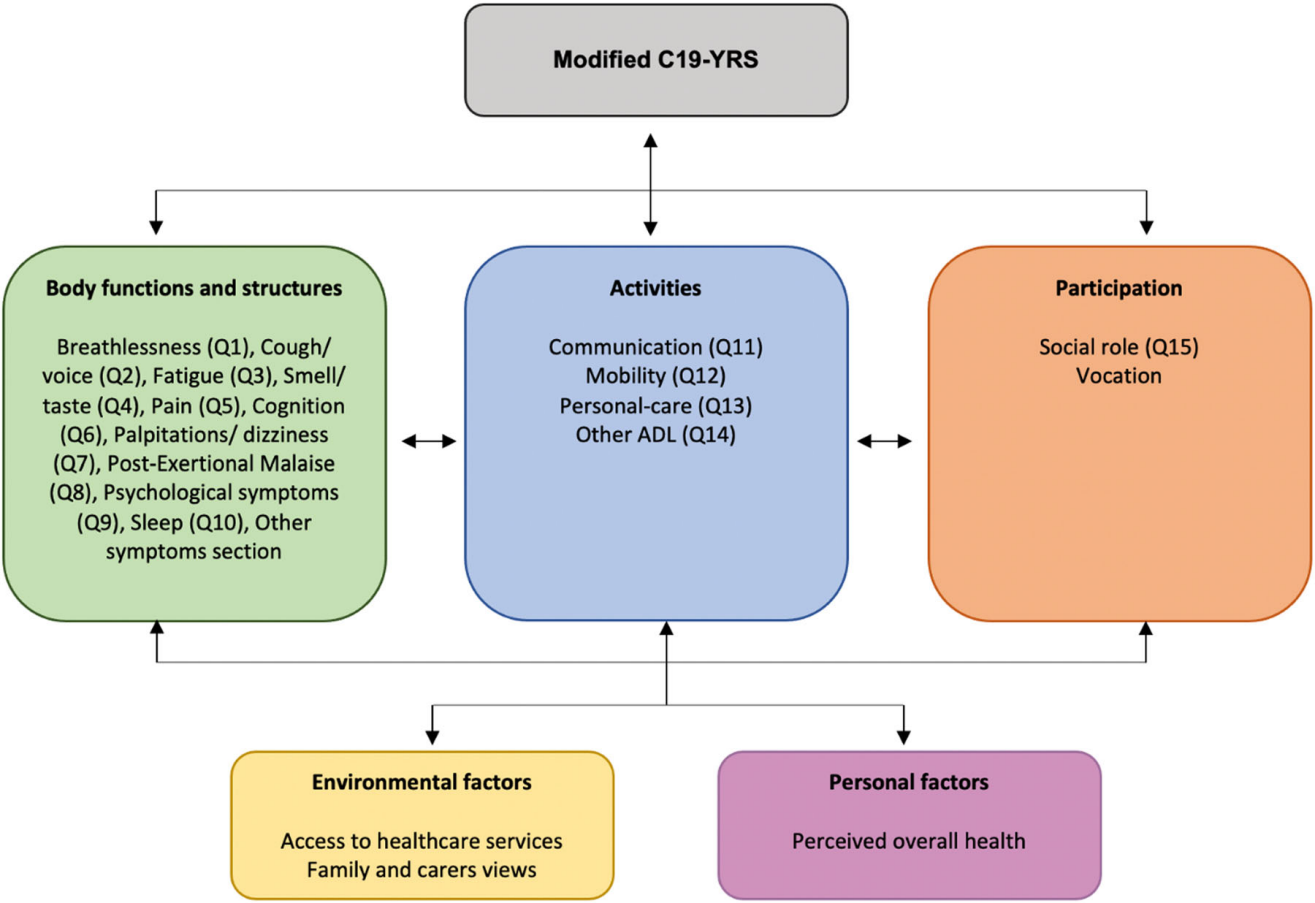
Mapping of the C19‐YRSm onto the WHO ICF framework

The WHO's International Classification of Functioning, Disability and Health (ICF) provides a framework for understanding the relationship between different aspects of any health condition.^6^ The domains covered by the C19‐YRSm, when mapped to the components of ICF (Figure 5), show that there is satisfactory capture of all the components (body functions and structures, activities, participation, environmental factors, and personal factors), making it suitable for a comprehensive biopsychosocial assessment of the condition.

Our future work with the scale will involve further evaluation of psychometric properties and validation of the C19‐YRSm in the Long Covid population. The NIHR‐funded project Long Covid Multidisciplinary Consortium for Optimising treatments and services acrOss the NHS (LOCOMOTION) is a platform of >5000 patients in the UK whose symptoms and functional limitations will be captured using C19‐YRSm at regular 3‐monthly intervals.^26^ We will have the opportunity to assess the construct and criterion validity of the scale, responsiveness, and ability to monitor the effectiveness of interventions, along with picking up the natural daily and weekly fluctuations of the condition. This more extensive data set can also estimate how effectively the measure captures differences between individuals and changes over time within the same individual. The floor and ceiling effects of the scale will be assessed to establish the dynamic measurement range of the scale, and we will estimate how effectively the measure captures the slight differences between individuals along the clinical spectrum of the condition.

We will also evaluate the respondent burden of completing the measure within the population. We will assess the use of digital tools, which can be challenging in certain cohorts (such as those with cognitive problems and those who do not use smartphones). The C19‐YRSm will undergo further Rasch analysis to validate the scale and determine its validity as an outcome measure in PCS. Additionally, when the assumptions of the Rasch model are satisfied, it is possible to transform the ordinal‐level scale raw scores to an interval‐level score, due to the sufficiency of the raw score.^27^ This was not the aim of the current project, but a large‐scale validation project of the C19‐YRSm would allow for creating a stable interval‐level transformation table.

The C19‐YRSm has an advantage over individual symptom‐specific measures used in PCS studies. It is comprehensive in covering most symptoms, less burdensome, and condition‐specific (compared to symptom‐specific measures that have been developed for other conditions).^28^ There is also an opportunity to explore whether C19‐YRSm could be developed into a preference‐based scale and undertake an economic evaluation of resource use and QALY analysis. The findings of this economic evaluation research are likely to influence local policy, commissioning, and service delivery that is needed to manage the growing number of Long Covid cases worldwide.

The proposed C19‐YRSm is a 17‐item PROM, with each item rated on a 0–3 numerical rating scale. Zero represents symptom not present, 1 represents a mild problem (not affecting daily life), 2 moderate problem (affecting daily life to a certain extent), and 3 illustrates severe problem (life disturbing or affecting all aspects of daily life). The C19‐YRSm, similar to the original version, is broken down into four subscales concerned with the severity of patients' key symptoms, functional limitations, other symptoms, and overall health. Pre‐COVID scores are also captured for comparison (see Supporting Information: Appendix I). The worst scores for each item (Questions 1–10) form the symptom severity subscale (score 0–30), Questions 11–15 the functional disability subscale (0–15), Question 16 is the other symptoms subscale (0–25), and Question 17 is the overall health score (0–10).

This study has some limitations. Firstly, the new scale response categories were done post hoc on data collected from the original version of C19‐YRS. Therefore, future studies need to undertake Rasch analysis to test the psychometric properties of prospectively collected data on the new scale (C19‐YRSm). PCS is a fluctuant condition, and symptoms vary between days and weeks. This cross‐sectional study was not designed to capture such fluctuations. Future longitudinal studies need to explore whether C19‐YRSm is sensitive to capture the remitting relapsing nature of the condition. Finally, there will be some uncommon symptoms and functional limitations that are not captured by the scale. Such aspects should be captured under the “other symptoms” section of the scale in future studies (such as the LOCOMOTION study)^26^ informing the future development of the scale.

To conclude, a condition‐specific patient‐reported outcome measure, C19‐YRSm, has been developed to capture the common symptoms, functional disability, and overall health in Long Covid. The scale content covers all aspects of the WHO ICF framework. The scale allows patients and health care staff to monitor these aspects over the course of the condition, potentially capture Long Covid fluctuations and assess the impact of rehabilitation interventions for the condition.

### 4.1 Using the scale

The C19‐YRSm is free to use (Supporting Information: Appendix I), and the MS Word/PDF copy of the tool is available on the University of Leeds website. The digital PROM system developed by ELAROS comprises a smartphone application for the patient and a web portal for the clinicians managing the patient's care. The digital system has C19‐YRSm and other scales used in PCS care and is currently being used in more than 30 NHS Trusts in the UK. Any clinical service worldwide wishing to acquire the digital system can contact ELAROS, who will demonstrate the system and provide necessary training to the system's users.

University of Leeds and the authors hold the copyright for the scale. The scale will remain free for use. Any organization wishing to administer the scale to patients for a charge or add the scale to a commercial digital platform should contact the University of Leeds or the corresponding author to seek the required approvals.

## AUTHOR CONTRIBUTIONS

Manoj Sivan is the project lead and conceptualized the study. Manoj Sivan, Amy Parkin, Stephen Halpin, Denise Ross, and Mike Horton obtained institutional approvals. Amy Parkin, Sophie Makower, Mike Horton, Denise Ross, and Manoj Sivan developed the data collection tool and gathered data. Mike Horton and Nick Preston undertook the Rasch analysis. All authors contributed to the working group involved in developing the C19‐YRSm. Manoj Sivan and Mike Horton wrote the first draft of the manuscript, and all authors revised the manuscript. All authors read and approved the final manuscript.

## CONFLICT OF INTEREST

The authors declare no conflict of interest.

## ETHICS STATEMENT

Approvals were obtained from the University of Leeds and Leeds Community Healthcare NHS Trust for evaluation and secondary analysis of C19‐YRS data. All participants consented for their data to be used for evaluation and research purposes.

## Supporting information

Supporting information.

## Data Availability

The datasets used and analyzed during the current study are available from the corresponding author on reasonable request.
